# Photo-thermal effect enhances the efficiency of radiotherapy using Arg-Gly-Asp peptides-conjugated gold nanorods that target αvβ3 in melanoma cancer cells

**DOI:** 10.1186/s12951-015-0113-5

**Published:** 2015-08-28

**Authors:** Ping Li, Yi-wen Shi, Bing-xin Li, Wen-cai Xu, Ze-liang Shi, Chuanqing Zhou, Shen Fu

**Affiliations:** Department of Radiation Oncology, Shanghai Proton and Heavy Ion Center, Fudan University Cancer Hospital, Shanghai, People’s Republic of China; School of Biomedical Engineering, Shanghai Jiao Tong University, Shanghai, People’s Republic of China; Department of Radiation Oncology, 6th People’s Hospital of Jiao Tong University, Shanghai, People’s Republic of China; Department of Radiation Oncology, Zhengzhou University He’nan Cancer Center, Zhengzhou, He’nan People’s Republic of China

**Keywords:** Arg-Gly-Asp peptides, Gold nanorods, Integrin αvβ3, Photo-thermal therapy, Radiotherapy

## Abstract

**Background:**

Thermotherapy has been known to be one of the most effective adjuvants to radiotherapy (RT) in cancer treatment, but it is not widely implemented clinically due to some limitations, such as, inadequate temperature concentrations to the tumor tissue, nonspecific and non-uniform distribution of heat. So we constructed arginine-glycine-aspartate peptides-conjugated gold nanorods (RGD-GNRs) that target the alpha(v) beta(3) Integrin (αvβ3) and investigate whether the photo-thermal effect of RGD-GNRs by near infrared radiation (NIR) could enhance the efficiency of RT in melanoma cancer cells.

**Results:**

RGD-GNRs could be seen both on the surface of the cell membranes and cytoplasm of A375 cells with high expression of αvβ3. After exposed to 808 nm NIR, RGD-GNRs with various concentrations could be rapidly heated up. Compared to other treatments, flow cytometric analysis indicated that RT + NIR + RGD-GNRs increased apoptosis (*p* < 0.001) and decreased the proportion of cells in the more radioresistant S phase (p = 0.014). Treated with NIR + RGD-GNRs, the radiosensitivity was also significantly enhanced (DMF_SF2_: 1.41).

**Conclusion:**

Results of the current study showed the feasibility of using RGD-GNRs for synergetic RT with photo-thermal therapy. And it would greatly benefit the therapeutic effects of refractory or recurrent malignant cancers.

## Background

Radiotherapy is an effective treatment for numerous cancers. It is estimated that radiotherapy contributes approximately 40 % towards curative treatment of cancer patients [1]. However, radiotherapy is mainly effective to G2/M phase tumor cells, with little effect to hypoxic cells and S phase cells, which is the major reasons for recurrence and deadly metastasis after radiotherapy. Thermotherapy, as a promising approach for killing radioresistant cancer, has been known to be one of the most effective adjuvants to radiotherapy. In addition to direct toxic effect on tumor cells, hyperthermia can kill radioresistant hypoxic cells and S phase cells [2]. Some pre-clinical and clinical researchers showed synergetic effects of combining radiotherapy and thermotherapy, especially for refractory or recurrent malignant tumors [3–5]. However, the use of this combined therapy approach has not been adopted in routine clinical practice. This can probably be attributed to the limitations of thermotherapy, such as inadequate temperature concentrations to the deep tumor tissue, nonspecific and non-uniform distribution of heat [6].

To overcome these obstacles, researchers have introduced the use of nanotechnology to thermo-radiotherapy [7]. Gold nanorods (GNRs) are ideally suited for photo-thermal therapy because of their unique optical characteristics [8, 9]. In particular, when light of wavelength in the visible to near infrared radiation (NIR) region interacts with the particles much smaller than the incident wavelength, the electric field of the light wave induces coherent oscillation of the free electrons locally around the nanoparticle, with a frequency known as the localized surface plasmon resonance frequency [10]. It is easy to tune the longitudinal surface Plasmon resonance (LSPR) wavelengths of GNRs through chemical methods, so that they can match with the center wavelengths of the laser [11]. GNRs of defined size can absorb NIR, leading to the temperature rise over 40 °C. Such temperature is sufficient for sensitizing tumor cells to radiotherapy [12]. Furthermore, GNRs could be used as radiosensitizers due to the high atomic (Z) number elements (Au) [13]. Gold nanoparticles have been shown to cause sensitization with kilovoltage (KV) or Megavoltage (MV) X-ray in vitro and in vivo studies [14, 15]. We have previously shown that GNRs could sensitize melanoma cells to clinically relevant 6MV X-ray irradiation [15]. Moreover, by conjugating some specific antibodies and peptides, the amount of GNRs accumulated in the tumor site raises greatly, thus enhancing thermal and radio efficacy.

One of such molecular targets is integrins. Over-expression of integrins has been reported in a wide variety of tumors, such as breast cancer, melanoma, and glioblastoma [16]. And it is approved that over-expression of integrins is associated with tumor growth, metastasis and angiogenesis [17]. Among all the integrins, alpha(v) beta(3) Integrin (αvβ3) is involved in tumor metastasis, angiogenesis, as well as radiosensitivity. Therefore, targeting αvβ3 has attracted a great deal of attention due to its role as a potential target for inhibition of angiogenesis and tumor growth. Arginine (R)-glycine (G)-aspartate (D) (RGD) is the most effective and widely used peptide sequence for stimulating cell adhesion on synthetic material surfaces. RGD molecular probes have been developed for selectively binding to αvβ3. Once the RGD sequence is recognized and bound to integrins, it initiates an integrin-mediated cell adhesion process and activates signal transduction between the cell and extracellular matrix [18].

In this study, we synthesized multifunctional RGD (Arg-Gly-Asp peptides)-conjugated gold nanorods (RGD-GNRs). Such RGD-GNRs could produce significant thermal effect upon 808 nm NIR and simultaneously serve as radiosensitizers by the high Z gold. RGD served as a targeting probe to specifically conjugate to tumor cells and we compared the effect of combined treatment of photo-thermal therapy and radiotherapy with either alone. As a result, the combined treatment exhibited a synergistic effect.

## Results and discussion

### Characterization of GNRs

**Fig. 1:**
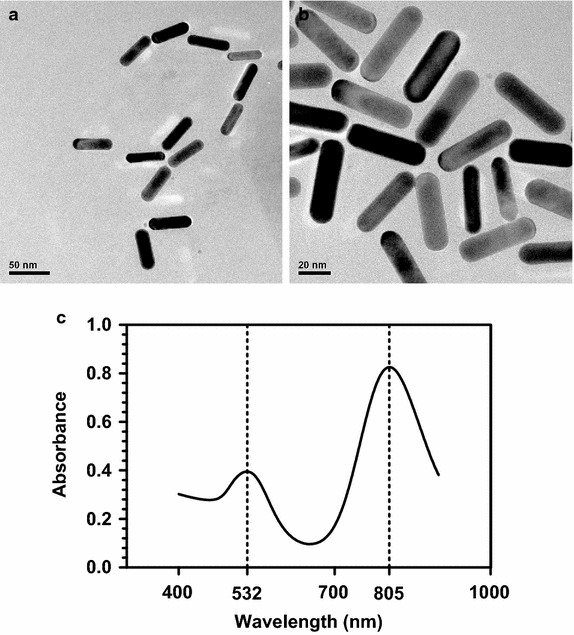
**a** The TEM images of GNRs dispersed in PBS. *Scale bar* 50 nm. **b** The TEM images ofGNRs dispersed in PBS. *Scale bar* 20 nm. **c** Measured UV–VIS absorbance spectrum of the synthesized GNRs, showing peak extinction at 805 and 532 nm, respectively.

Before carrying out our studies, we synthesized and characterized the GNRs. The TEM image and measured UV–VIS spectrum are shown in Fig. 1. The length of the GNRs was about 44.4 nm and the width 15.6 nm (Fig. 1a, b). As shown in Fig. 1c, the LSPRs of GNRs located at 805 nm. In our nanoconstruct, RGD peptide with high αvβ3 binding affinity and low cytotoxicity was conjugated to GNRs through a PEG linker (RGD-GNRs) [15].

### RGD mediated specific cell uptake of RGD-GNR

**Fig. 2:**
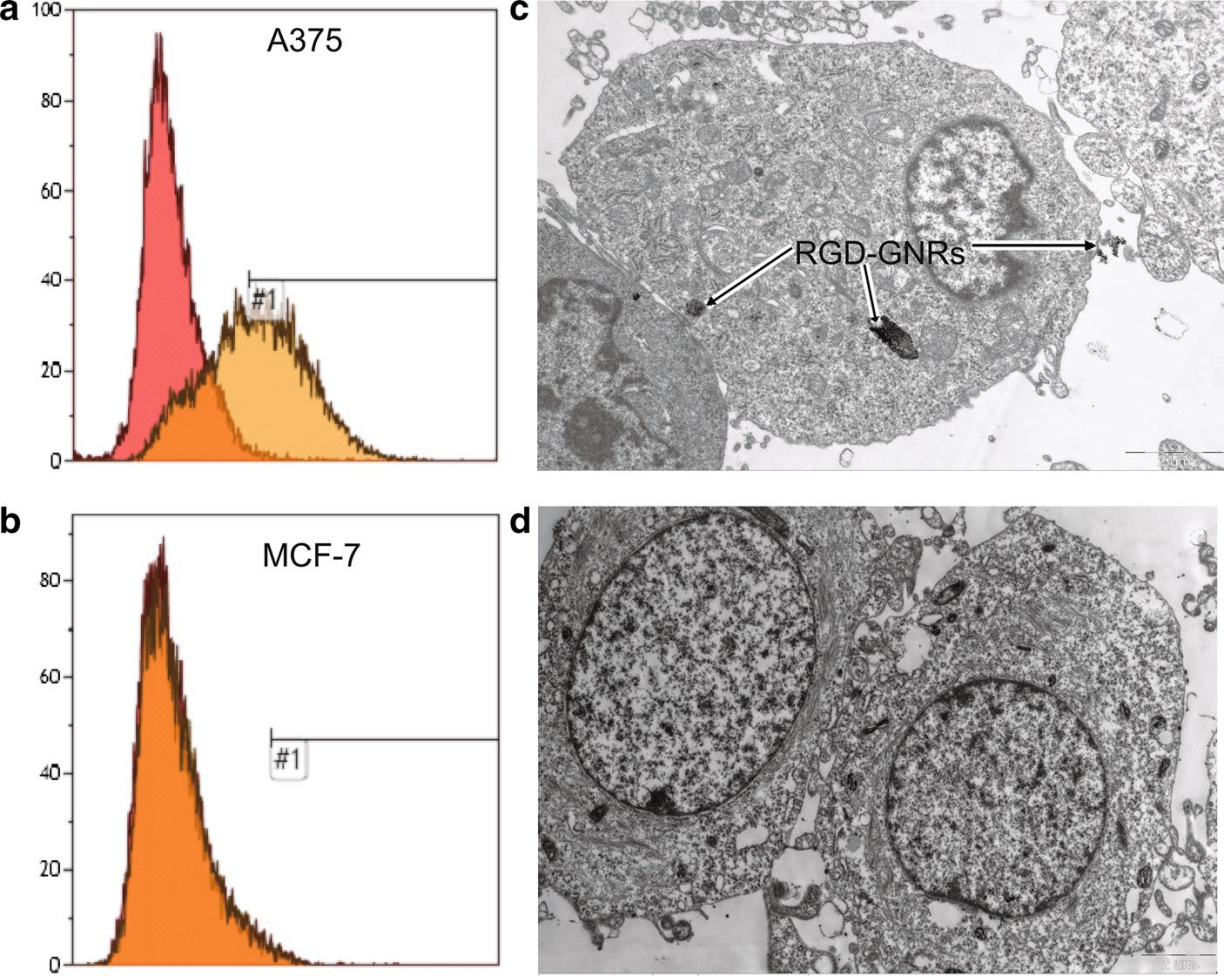
Cellular uptake of RGD-GNRs. **a**, **b** Representative photos of flow cytometry assay of the expression level of αvβ3 in A375 and MCF-7 cells. **c**, **d** TEM analysis of A375 (**c**) and MCF-7 cells (**d**) with and without internalized RGD-GNRs.

Next, we evaluated the selectivity of uptaking RGD-GNRs in αvβ3 positive tumor cells. A375 melanoma cancer cells and MCF-7 breast cancer cells with different expression of αvβ3 were chosen. Expression of αvβ3 in these cell lines was investigated by flow cytometry. As shown in Fig. 2a, b, there was a significantly higher expression level of αvβ3 in A375 cells but lower expression level of that in MCF-7 cells. Meantime, significantly more RGD-GNRs were internalized in A375 cells than in MCF-7 cells (Fig. 2c, d). These results indicated that cell uptake of RGD-GNRs were mediated by αvβ3.

### Photo-thermal effect caused by GNRs under NIR irradiation

**Fig. 3:**
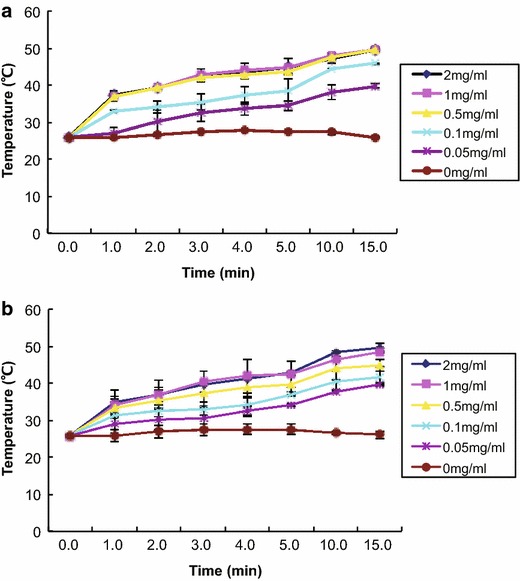
Heating curve of different concentrations of GNRs (**a**) and RGD-GNRs (**b**) (0, 0.05, 0.1, 0.5, 1, 2 mg/ml) under 808 nm NIR at a power density of 1 W/cm^2^.

As is known to all, GNRs exhibit surface plasmon resonance, allowing them to absorb far stronger light in near-infrared area (650–900 nm) [8, 9]. Since it is easy to tune the LSPR wavelengths of GNRs, they can match the center wavelength of NIR laser source in photo-thermal treatment. In this study, the LSPR wavelength of GNR locates at 805 nm. In order to verify the potential of GNRs as the photo-thermal agent, GNRs and RGD-GNRs at different concentrations were exposed to 808 nm NIR at a power density of 1 W/cm^2^ for 15 min. Figure 3 showed the h eating curve of GNRs and RGD-GNRs at different concentrations. It was shown that after exposure to the NIR, each concentration of GNRs or RGD-GNRs rapidly warmed within 1 min. An obvious concentration-dependent temperature increase was observed either in GNRs or in RGD-GNRs, After 15 min of continuous NIR irradiation at 1 W/cm^2^, the temperature of GNRs or RGD-GNRs group increased about 13 °C when the concentration was 0.05 mg/ml and the growth of temperature was of a concentration-dependent manner. As expected, there was no temperature change at all in the groups that did not treated with RGD-GNRs followed by NIR irradiation. The above results well demonstrate the suitability of RGD-GNRs as efficient radiosensitizers and photo-thermal agents.

### Photo-thermal effect enhanced radiosensitivity in A375 cells

Phase II/III clinical trials have demonstrated that hyperthermia combined with radiotherapy is beneficial for tumor control and survival in patients with radioresistant tumors of different types [4]. Based on the clinical experience, we further assessed the synergistic effect between radiotherapy and photo-thermal therapy. Survival fraction at 2 Gy (SF2) and dose-modifying factor (DMF) values were used to quantify the radiosensitizing effect of the cells. DMF_SF2_: SF2 (radiation alone)/SF2 (radiation + treatment). We treated the A375 cells with RGD-GNRs by 808 nm NIR irradiation for 15 min combined with RT. It was found that treatment of A375 cells with RGD-GNRs resulted in increased radiosensitivity with DMF_SF2_ = 1.288; however, treatment with NIR was not effective (DMF_SF2_ = 1.022). The radiosensitizing effect was further enhanced by combination treatment with RGD-GNRs and NIR, with a DMF_SF2_ of 1.413. These results confirmed that RGD-GNRs combined with NIR enhanced the radiosensitizing effect by photo-thermal effect.

### Enhanced radiation-induced apoptosis by photo-thermal treatment

**Fig. 4:**
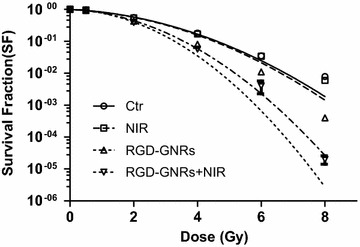
Radiosensitizing effect by RGD-GNRs and/or NIR. A375 cells were exposed with DMEM (control group), NIR, RGD-GNRs (50 μg/ml) or RGD-GNRs + NIR for 1 h, then irradiated at 0–8 Gy with 6MV-X ray. Cells were trypsinized, counted, and seeded at different dilutions. Colonies of 0.50 cells were counted approximately 2 weeks after treatment.

To identify the effect of photo-thermal treatment combined with radiotherapy on apoptosis, we evaluated the apoptosis level based on the flow cytometry. As shown in Fig. 4, NIR or RGD-GNRs alone did not significantly increase the amount of cell apoptosis compared with the control group (NIR vs. Ctr, *p* = 0.478; RGD-GNRs vs. Ctr, *p* = 0.064). Radiation alone or combined with NIR slightly enhanced apoptosis compared with control group (RT vs. Ctr, p = 0.007; RT and NIR vs. Ctr, *p* = 0.002). The combination of NIR and RGD-GNR with RT resulted in increased apoptosis compared with other treatment groups (*p* < 0.001). Taken together, these data confirmed the synergistic interaction between radiotherapy and photo-thermal treatment.

### Decrease the proportion of cells in S phase by photo-thermal treatment

**Fig. 5:**
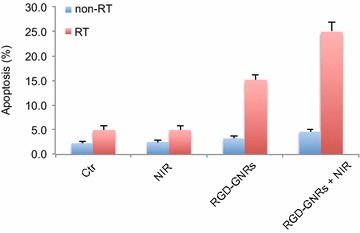
The apoptosis of the A375 cells after NIR or/and RGD-GNRs treatment. A375 cells were treated with RGD-GNRs or/and irradiation for 1 h prior to irradiation (6 mV X-rays with a dose of 4 Gy). The cells were stained with Annexin V and propidium iodide, and apoptosis was analyzed by flow cytometry after 24 h of treatment.

**Fig. 6:**
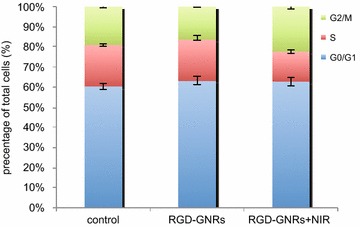
The changes in cell cycle distribution by treatment of RGD-GNRs and NIR in A375 cells. A375 cells were incubated with 0.05 mg/ml RGD-GNRs, then the cells were or were not irradiated with 808 nm NIR with the power density of 1 W/cm^2^ for 1 h. After 24 h, the cells were analyzed by flow cytometry.

Considering that hyperthermia-induced radiosensitization is known to be associated with more sensitive to S phase cells, which are resistant to radiation. It was determined whether a cell cycle rationale could be used to account for the greater efficacy of the photo-thermal treatment and radiotherapy combination (Fig. 5). As shown in Fig. 6, after treatment with RGD-GNRs and NIR for 24 h, there were a significant accumulation of cells in G2/M phase (*p* = 0.022, compared with other treatment groups) and a drastic decrease of S phase cells (*p* = 0.014, compared with other treatment groups), No significant difference can be seen in G0/G1 phase cells among the three groups. So hyperthermia may sensitize cells to radiation by regulating the cell cycle. The combination of thermotherapy and radiotherapy increases damage to all phases of tumor cells, but the killing effects of the S-phase cells are even more markedly enhanced. In addition to directly modulating the cell cycle, thermotherapy can also affect the radiosensitivity in another way. Hyperthermia leads to the denaturation of some proteins, including a variety of DNA-repair enzymes, resulting in irreversible damage [19].

## Conclusion

In summary, we showed that RGD-GNRs significantly enhanced radiosensitivity in A375 cells with high expression of αvβ3. Moreover, RGD-GNRs showed greater radiosensitizating effect when combined with NIR irradiation then RGD-GNRs alone. The significantly enhanced radiosensitivity of RGD-GNRs could be attributed to: (1) RGD-GNRs actively targeted to cancer cells because of high expression of αvβ3. (2) Potential effectiveness of GNRs as radiosensitizers for not only KV X-ray but also MV X-ray. (3) The energy of NIR could be absorbed by RGD-GNRs and transferred into local thermal energy, making RGD-GNRs as an ideally candidates for photo-thermal treatment. (4) The synergistic interaction between radiotherapy and thermotherapy. Currently, although extensive preclinical reports are available on the utility of gold nanoparticle for thermally therapeutic applications, there are few reports on using these features to improve the efficacy of radiotherapy. A potential limitation of this combined radio-photothermal treatment strategy is penetration depth of NIR irradiation. But, melanoma cancer, used in present study, is a superficial radioresistant malignant tumor, which is best suitable for the NIR induced photo-thermal treatment. To our knowledge, this is the first report that describes use of the RGD conjugated GNRs induced hyperthermia as an adjuvant for radiotherapy to treat melanoma cancer. Our results provide an interesting area for future radio-thermal therapy by some novel materials, such as nanoparticle, because it opens up new possibilities in cancer therapy.

## Methods

### Synthesis and characterization of RGD-conjugated gold nanorods

The gold nanorods were synthesized using the seed-mediated template-assisted protocol [20] by reducing gold salt in the presence of surfactant-directed synthesis. The seed solution was produced by the reduction of chlorozuric acid (5 ml, 0.5 mmol) with ice-cold NaBH4 (0.6 ml, 10 mmol) in the presence of CTAB (5 ml, 0.2 mol) solution (Sinopharm Chemical Reagent Co., Ltd. Shanghai, China). For the growth solution, 100 ml of 0.1 mol CTAB was first mixed with 0.01 mol AgNO_3_, followed by 0.01 mol chloroauric acid, 0.1 mol AA, 0.5 mol H_2_SO_4_, and 250 μl of seed solution. Finally, the resulting solution was incubated at 30 °C over a period of 20 h. The procedure of preparing RGD conjugated GNRs is introduced as follows. First, the as-prepared GNRs solution was centrifuged twice at 9,600 rpm for 25 min. Then, a stock solution of SH-PEG-COOH (10 mg/ml) (Shanghai Yare Biotech, Inc. Shanghai, China) was added into a freshly prepared GNR solution under vigorous stirring for 2 h. All the GNRs samples were centrifuged twice and redispersed in deionized water. The RGD peptides (China Peptides Co., Ltd. Shanghai, China) were covalently attached to the outer ends of the PEG-GNRs via amide bonds, using the standard EDC-NHS reaction [21]. A solution of RGD in deionized water was added to a volume of PEGylated gold nanorods to react for 3 h and excess RGD peptides were removed by centrifugation at 10,000 rpm for 10 min. The RGD-GNRs were dispersed in Dulbecco’s Modified Eagle Medium (Hyclone, Carlsbad, CA, USA) and stored at 4 °C for later cell experiments. GNRs samples were analyzed with a transmission electron microscopy (TEM) (JEM-2010HT, JEOL, Japan) operating at an accelerating voltage of 200 kV. UV–VIS absorbance spectra were measured with a UV–VIS spectrophotometer (Cary-100, Agilent, America) in the wavelength range from 400 to 900 nm.

### Cell lines and culture

Human melanoma A375 cells with high expression of αvβ3 and human breast cancer MCF-7 cells with low expression of αvβ3 were purchased from the Shanghai Institute of Cell Biology and Chinese Academy of Sciences (Shanghai, China) and grown in 5 % CO_2_ incubator at 37 °C using Dulbecco’s Modified Eagle Medium (DMEM) with 10 % fetal bovine serum (Hyclone).

### Integrin αvβ3 analysis

A375 and MCF-7 cells were collected and suspended in phosphate- buffered saline/0.2 % bovine serum albumin. A 200 μl sample of the suspension was incubated with a mouse antihuman integrin αvβ3 mAb LM609 (Millipore, Shanghai, China) or isotype-matched control antibody DD7 (Millipore) for 45 min at 4 °C. The cells were washed three times with phosphate-buffered saline and analyzed using a flow cytometry (Becton–Dickinson, Frank- lin Lakes, NJ, USA).

### TEM analysis of cells with internalized gold nanorods

A375 and MCF-7 cells previously incubated with RGD-GNRs were washed three times with phosphate-buffered saline and fixed with 2.5 % glutaraldehyde for 6 h. The cells were post-fixed in 1 % osmium tetroxide for 2 h, dehydrated in ethanol, and embedded in agar resin (Agar Scientific, Stansted, Essex, UK). Thin Sects. (60–70 nm) were collected on copper grids, stained with methanol and lead citrate, and analyzed with a analyzed with a TEM (JEM-2010HT, JEOL, Japan) operating at an accelerating voltage of 200 kV.

### Measurements of heating GNRs and RGD-GNRs by near-infrared radiation

The photo-thermal properties of gold nanorods were examined using a high-power NIR laser source. Laser irradiation was accomplished using an Connet Highly Stabilized Laser Source System (VLSM-808-B-070), with a wavelength of 808 nm at a power density of 1 W/cm2 and a spot size of 5 mm diameter. The GNRs or RGD-GNRs were diluted in DMEM with various concentrations. After ultrasonic dispersion, a 500 μl volume of gold nanorods with different concentrations were put into 600 μl EP tubes. Subsequently, these samples were irradiated with an NIR laser for 15 min. Meanwhile, the temperature was measured and the heating curves were drawn.

### Clonogenic assay

Exponentially growing cells were exposed with DMEM (control group), NIR, RGD-GNRs (50 μg/ml) or RGD-GNRs + NIR for 1 h, then irradiated at 0–8 Gy with 6MV-X ray. After 48 h, the cells were detached, counted, and seeded at different dilutions according to the irradiation dose and grown for 2 weeks to form colonies. The colonies were stained with 0.4 % crystal violet. Only colonies containing ≥50 cells were scored. All experiments were repeated three times. Survival curves were fitted to a linear quadratic (LQ) model using Graphpad Prism software (version 5.0).

### Cell cycle and apoptosis assays by flow cytometry

A375 cells (10^6^ cells/ml) fixed in 95 % ethanol at −20 °C for 24 h were washed with cold phosphate-buffered saline, resuspended, and stained with propidium iodide (50 μg/ml phosphate-buffered saline; Invitrogen, Shanghai, China) for 15 min at 4 °C. Analysis was performed using a FACS Calibur flow cytometry. Cellular DNA content and cell cycle data were analyzed by flow cytometry using multicycle system 2.0 software. For the apoptosis assay, the cells were stained with Annexin-V-fluorescein isothiocyanate/propidium iodide (Invitrogen) and measured by flow cytometry. All tests were repeated three times.

### Statistical analysis

All the experiments were completed in three triplicates, Statistical analysis was performed using Student’s t test, and p value was calculated based on two-tailed test. p < 0.05 was considered statistically significant. SPSS 11.0 software was used for all statistical analyses.

## Abbreviations

RGD: arginine (R)-glycine (G)-aspartate (D)
GNRs: gold nanorods
RT: radiotherapy
NIR: near infrared radiation
RGD-GNRs: arginine-glycine-aspartate peptides-conjugated gold nanorods
Ctr: control
LSPR: localized surface plasmon resonance
KV: kilovoltage
MV: Megavoltage
TEM: transmission electron microscopy
αvβ3: alpha(v) beta(3) Integrin
DMF_SF2_: DMF = dose-modified factor, SF2 = survival fraction at 2 Gy
DMEM: Dulbecco modified Eagle medium
